# Prevalence of relevant early complications during the first 24 h on a normal ward in patients following PACU care after medium and major surgery: a monocentric retrospective observational study

**DOI:** 10.1007/s00423-024-03480-z

**Published:** 2024-09-30

**Authors:** Anouk Wurth, Thilo Hackert, Dittmar Böckler, Manuel Feisst, Sabine Haag, Markus A. Weigand, Thorsten Brenner, Thomas Schmoch

**Affiliations:** 1 Department of Anesthesiology and Intensive Care Medicine, University Hospital Essen, University Duisburg–Essen, Hufelandstraße 55, 45147 Essen, Germany; 2 Department of Anesthesiology, Heidelberg University Hospital, Heidelberg, Germany; 3 Department of General, Visceral and Transplantation Surgery, University of Heidelberg, Heidelberg, Germany; 4 Department of General, Visceral and Thoracic Surgery, University Hospital Hamburg–Eppendorf, Hamburg, Germany; 5 Department of Vascular Surgery and Endovascular Surgery, Heidelberg University Hospital, Heidelberg, Germany; 6 Institute of Medical Biometry, University of Heidelberg, Heidelberg, Germany; 7 Department of Anesthesiology and Intensive Care Medicine, Hôpitaux Robert Schuman – Hôpital Kirchberg, Luxembourg City, Luxembourg

**Keywords:** Post-operative care unit, PACU stay, Early complications, Medium surgery, Major surgery

## Abstract

**Purpose:**

Even today, it remains a challenge for healthcare professionals to decide whether a clinically stable patient who is recovering from uncomplicated medium or major surgery would benefit from a postoperative intensive care unit (ICU) admission, or whether they would be at least as adequately cared for by a few hours of monitoring in the post-operative care unit (PACU).

**Methods:**

In this monocentric retrospective observational study, all adult patients who (I) underwent medium or major surgery between 1 January 1 2014 and 31 December 2018 at the Heidelberg University Surgical Center, and (II) were monitored for 1–12 h in the PACU, and then (III) transferred to a normal ward (NW) immediately thereafter were included. At the end of the PACU stay, each patient was cleared by both a surgeon and an anesthesiologist to be transferred to a NW. The first objective of this study was to determine the prevalence of relevant early complications (RECs) within the first 24 h on a normal ward. The secondary objective was to determine the prevalence of RECs in the subgroup of included patients who underwent partial pancreaticoduodenectomy.

**Results:**

A total of 10,273 patients were included in this study. The prevalence of RECs was 0.50% (confidence interval [CI] 0.40–0.60%), with the median length of stay in the PACU before the patient’s first transfer to a NW being 285 min (interquartile range 210–360 min). In the subgroup of patients who underwent partial pancreaticoduodenectomy (*n* = 740), REC prevalence was 1.1% (CI = 0.55–2.12%).

**Conclusion:**

Based on a medical case-by-case assessment, it is possible to select patients who after a PACU stay of only up to 12 h have a low risk of emergency readmission to an ICU within the 24 h following the transfer to the NW. Continued research will be needed to further improve transfer decisions in such low-risk subgroups.

**Supplementary Information:**

The online version contains supplementary material available at 10.1007/s00423-024-03480-z.

## Introduction

In Germany as well as in other European countries, in the United States of America and Japan, there are current recommendations for the organization of postoperative monitoring [1–6]. However, none of these recommendations explicitly state how long after medium or major surgery a patient should remain in a monitoring area such as an intensive care unit (ICU), intermediate care unit (IMC), or post-operative care unit (PACU). It is recommended that patients should be discharged from the monitoring area to a normal ward (NW) at the physician’s professional discretion as supported by clinical scores [1–4]. When deciding on the best time to move patients to a NW, physicians must consider the requirements for delivering pain management and ensuring vital functions (such as airway control, respiration and and cardiocirculatory stability), and the stochastic risk of secondary bleeding plays a major role in this decision-making process [1–4].

Although data on current postoperative monitoring periods and protocols is scarce, many hospitals may use routine ICU-admission protocols to manage and reduce the risks of postoperative complications following medium or major surgery, such as pancreatic or liver surgery); ICU monitoring may be assumed to be mandatory at least for the first postoperative night after scheduled operations (and for comparable monitoring times for procedures performed at night or in the late evening) [7–11].

In contrast, patients at the Heidelberg University Hospital are only monitored for several hours in the PACU following uncomplicated medium and major surgical procedures. Internal planning usually schedules four to six hours (240–360 min), for the PACU stay, although this can be adapted to meet each patient’s needs. In cases of elective surgical procedures, which are usually performed in the morning, the aim is to transfer the patient to the normal ward (NW) on the same day. Generally, this means a stay in the PACU for monitoring for up to 12 h (≤ 720 min). In everyday practice, this procedure of monitoring stable patients for only a few hours is also extended to patients who are transferred to the PACU after elective operations or emergencies in the late afternoon or at night.

Despite there being evidence that ICU admissions following non-cardiac surgery are not associated with a survival benefit [12], to date there is little data supporting the safety of a fast-tracked approach such as that described above. Although it is undisputed that patients who are initially unstable after major surgery—for example, because they require circulatory support or ventilation—must remain in an ICU to be monitored, it remains unclear whether a routine stay in ICU for monitoring is required by patients with stable respiratory and cardiocirculatory functions who have a low risk of bleeding according to their surgeon. Therefore, our first aim was to describe the prevalence of short-term relevant early complications (RECs) leading to ICU admission among this specific subset of post-operative patients, who had stayed in a PACU for up to 12 h following uncomplicated medium or major surgery, and had then been cleared both by their surgeon and an anesthesiologist for transfer onto a normal ward. The second aim of this study was to determine the prevalence of RECs among the high-risk subgroup of patients who underwent elective partial duodenopancreatectomy (the Whipple procedure). Further outcome parameters were hospital mortality, association of emergency status with REC prevalence, association of daytime of admission to the PACU and daytime of transfer from the PACU onto NWs with REC prevalence, and length of hospital-stay of patients following elective partial duodenopancreatectomy.

## Methods

### Study population

**Fig. 1:**
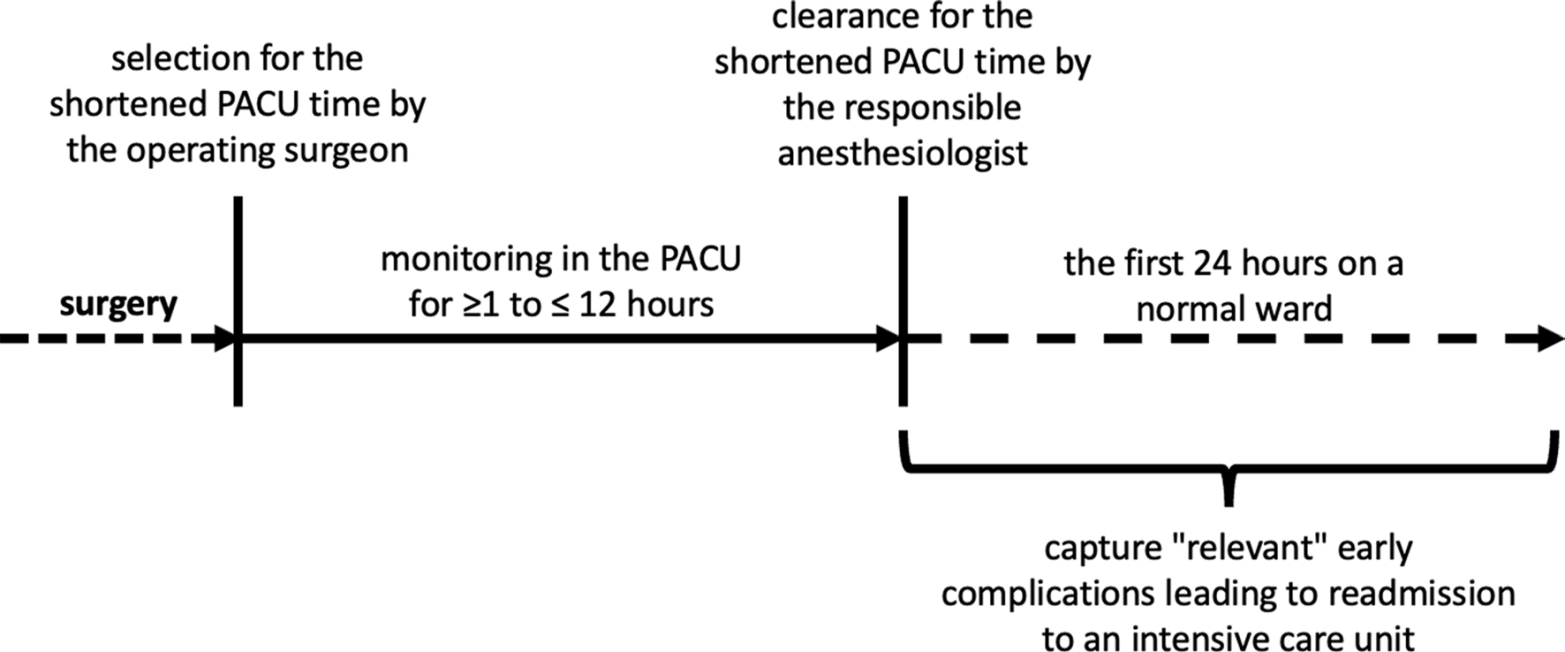
Selection of patients for a shortened post-operative stay in the PACU

All patients who underwent surgery at the Heidelberg University Surgical Center between 1 January 2014 and 31 December 2018, and whose surgery was conducted by the Department of General, Visceral, and Transplant Surgery; the Department of Vascular Surgery and Endovascular Surgery; or the Department of Urology at Heidelberg University Surgical Hospital, were potentially eligible for inclusion in the study. The remaining inclusion criteria for this study were as follows: (a) the patient’s surgery was in the medium- or high-risk category according to the European Society of Anesthesiology and Intensive Care (ESAIC) classification of surgical risks; (b) the patient received postoperative monitoring in the central PACU of the Department of Surgery at Heidelberg University Hospital; (c) the patient was at least (≥) 18 years old at the time of surgery; (d) surgical and anesthesiologic clearance had been given, to transfer the patient after ≤ 12 h (≤ 720 min) observational stay in the PACU onto a normal ward; (e) the patient stayed in the PACU 1–12 h (≥ 60 min and ≤ 720 min); and (f) the patient was directly transferred from the PACU to a NW within the Department of Surgery at Heidelberg University Hospital [13]. Patients could be included multiple times in the study, both during a single hospitalization and over the course of multiple hospitalizations. However, patients who were transferred to the NW for purely palliative care at the end of life were excluded from the study. Figure 1 illustrates the selection process followed for this study.

### Rationale for the analyzed PACU monitoring time

The minimum PACU stay time of 1 h (60 min) was selected because a patient is occasionally booked incorrectly into the PACU through the hospital information system (HIS). in a mistake that is usually corrected after only a few minutes. A one hour lower limit for PACU stay duration was selected to exclude incorrect PACU bookings and because it was considered unlikely that a patient would be monitored in the PACU for less than one hour after a medium- or high-risk operation. An upper time limit of 12 h (720 min) was chosen to include patients who were transferred to a NW after elective surgery (which is usually performed in the morning) on the day of surgery. Using this criterion, it was possible to include also patients who were admitted to the PACU after elective surgery in the late evening or at night or as emergencies.

### Surgical clearance

The responsible surgeon determined whether a patient was eligible for a shortened observational PACU stay based mainly on the course of the surgery, followed by the patient’s risk of bleeding. In general, a patient was considered to be suitable for a shortened PACU stay after a surgery such as an uncomplicated duodenopancreatectomy or a partial liver resection. In cases with extensive interventions or in cases where the surgeon suspected an increased risk based on the intraoperative findings, the patience was denied clearance to leave the PACU after less than 12 h, and the patient either remained in the PACU for extended monitoring or was admitted to the IMC or ICU; the patient then underwent daily stability reassessment to determine when they were ready for transfer to a NW). This was the case for all visceral surgical vascular resections and reconstructions, extensive multivisceral resections, solid organ transplantations, total pancreatectomies, Truncus coeliacus resections, partial vena cava resections, and Thoracic Endovascular Vascular Repairs or thromboendarterectomies (TEA) for symptomatic stenosis of the internal carotid artery.

### Anesthesiologic clearance

Patients were only transferred from the PACU after clearance for the transfer was given by an anesthesiology specialist or an anesthesiologist in at least the 4th year of advanced training. The clearance decision was a medical decision based on an overall assessment of the individual patient and, as such, was subjective. However, the criteria used were adapted from the modified Alderate score [14, 15], and the minimum requirements for transfer were detailed in a standard operating procedure (SOP; *Online Source List SI 1*). Compliance with the SOP was regularly (several times a day) verified by the Head of Department. In addition to the points listed in the SOP, most anesthesiologists checked liver values, pancreas values, creatinine levels, and coagulation parameters; In case of unusual changes in the laboratory values that exceeded the expected perioperative range, the PACU stay was extended. Likewise, in cases in which there were doubts about the patient’s stability or their ability to be adequately assessed, the patient’s stay in the PACU was extended. In this context, it is worth mentioning that neither the length of the operation, nor the ASA status, nor intraoperative blood loss were used as singular decision criteria to deny anesthesiologic clearance.

### Characteristics of the PACU

The PACU at Heidelberg University Hospital was open 24/7 during the observation period. It had 13 beds for patients, 8 of which could be used for invasive ventilation. The PACU was equipped as an ICU; one intensive care nurse was available for every two ventilated patients and for a maximum of four non-ventilated patients. In addition, the PACU was staffed 24/7 by an anesthesiologist.

### Characteristics of the ICU and IMC

The ICU is staffed and equipped to provide maximum intensive care therapy (including e.g. invasive ventilation, and extracorporeal membrane oxygenation). Likewise, monitoring, catecholamine therapy, dialysis, and non-invasive continuous positive airway pressure (CPAP) ventilation can be carried out on the IMC ward. However, the IMC ward is not able to provide invasive ventilation.

### Settings on NWs

Medical rounds took place once a day on the NWs. The nursing staff were experienced in dealing with patients after major operations and received continuing education in postoperative nutrition, pain management, and recognizing postoperative complications. Blood samples were collected every morning to monitor blood count, coagulation, electrolytes, and kidney function as well as liver and pancreas function. Vital signs—pulse, blood pressure and saturation—were routinely recorded three times a day and additionally as required. Urine excretion, bowel movements, and fluid losses via any inserted drainage tubes were documented daily. Patients could call a nurse at any time, and a surgeon on duty was available 24/7 if required. For potentially vital emergencies, there was a medical emergency team including an anesthesiologist. Catecholamine treatment, however, could not be continued on NWs.

### Definition of RECs

An REC was defined as a complication that met the following criteria: (a) it occurred within the first 24 h after the transfer from PACU to a NW *and* (b) it was so severe that the patient had to be transferred back to a monitoring area (PACU, ICU, IMC, or operating room) because of this complication or that the patient died within the first 24 h following the transfer to the NW.

### Data collection

Data was extracted from the hospital information system (HIS) “IS–H med™” system (SAP, Walldorf [16]). Surgical procedures were coded according to the Operation and Procedure Code of the International Classification of Procedures in Medicine (OPS) [17]. The date of surgery, date of birth, and principal diagnosis coded according to the “International Classification of Mortality and Morbidity Statistics – Tenth Revision” (ICD–10) were extracted from the HIS, as were any recorded secondary diagnoses [17, 18]. In addition, time of admission and times of patient movements from the PACU to the NW or transfers back to a monitoring area were traced. In addition, pre-existing conditions recorded preoperatively by an anesthesiologist (e.g. pre-existing renal insufficiency, diabetes mellitus, chronic obstructive pulmonary disease, sleep apnea syndrome, or heart disease); height; weight; urgency or emergency status of the procedure (surgery required immediately or within 2 h); and American Society of Anesthesiologists (ASA) classification were extracted from the HIS [19, 20]. It is worth noting that it was standard practice to have a recent electrocardiogram (ECG), pulmonary function diagnostics, and the results of a current blood test available at the time of the anesthesiology pre-op consultation; this included at least blood count, electrolyte, liver, pancreas, and kidney values and coagulation parameters. Based on these test results, the patient’s medical history was cross-checked by an anesthesiologist prior to surgery. Both the Revised Cardiac Risk Index (RCRI) and the Charlson Comorbidity Index (CCI) were calculated from the collected data. Moreover, for patients undergoing pancreatic surgery, the duration of the operation was extracted from the anesthesia protocol, the and length of hospital stay was collected from the HIS. In addition, hospital mortality was recorded for patients with RECs.

The RECs that were identified retrospectively in this manner were verified and categorized by two anesthesiology residents into one of the following categories: (1) postoperative hemorrhage, (2) cardiovascular complication, (3) pulmonary artery embolism, (4) ischemia of an extremity, (5) abdominal complication, (6) neurologic complication (stroke, delirium, seizure, or psychogenic), (7) respiratory complication, (8) acute renal failure, (9) sepsis, or (10) pain. All decisions regarding this categorization were unanimous.

### Determination of the cardiovascular risk category

Based on the main procedures coded in the HIS, each case was assigned a risk category according to the classification published in the joint paper of the European Society of Cardiology (ESC) and the [10]. As the joint paper of ESC and ESAIC refers to the work of Glance et al. [21], in cases of doubt, the categorization made by Glance and colleagues was consulted when assigning a risk category. In cases with contradiction between the categorization of Glance and colleagues and the categorization of the ESC/ESAIC working group, decisions were made according to the latter [13, 21].

### Ethics

The work presented here was carried out in accordance with the professional code of conduct for physicians of the Baden-Württemberg Medical Association and the Declaration of Helsinki [22, 23]. This retrospective study was approved by the institutional review board (IRB) of the Heidelberg Medical Faculty (vote: S–598/2019). Requirement for written informed consent was waived by the IRB, as only routine care data were evaluated. The analysis was performed by physicians in the Department of Anesthesiology of the Heidelberg University Hospital, who had access to the data in the course of providing medical care and were bound by medical confidentiality. The data were evaluated in a pseudonymized manner.

### Statistics

Detailed descriptive statistics were calculated for the collected data. Unless explicitly stated otherwise, median, and 25th–75th percentiles (interquartile range, IQR) or absolute and relative frequencies were reported. Descriptive p-values, depending on the underlying empirical distribution, were calculated using the Chi-square test or Mann-Whitney U test as appropriate. Confidence intervals for proportions were calculated using the hybrid Wilson/Braun method [24–26]. A p-value of less than 0.05 was assumed to be statistically significant. P-values were reported in the full knowledge that, due to the retrospective nature of the work, they have only descriptive character. Wherever appropriate, statistical graphs were created to visualize analysis results. Statistical analysis and graphical visualization were performed using PRISM 9 for Mac (version 9.3.1, GraphPad Software Inc., San Diego, CA, USA) and Microsoft© Excel for Mac (version 16.61.1, Microsoft Corporation, Redmond, WA, USA).

## Results

**Fig. 2:**
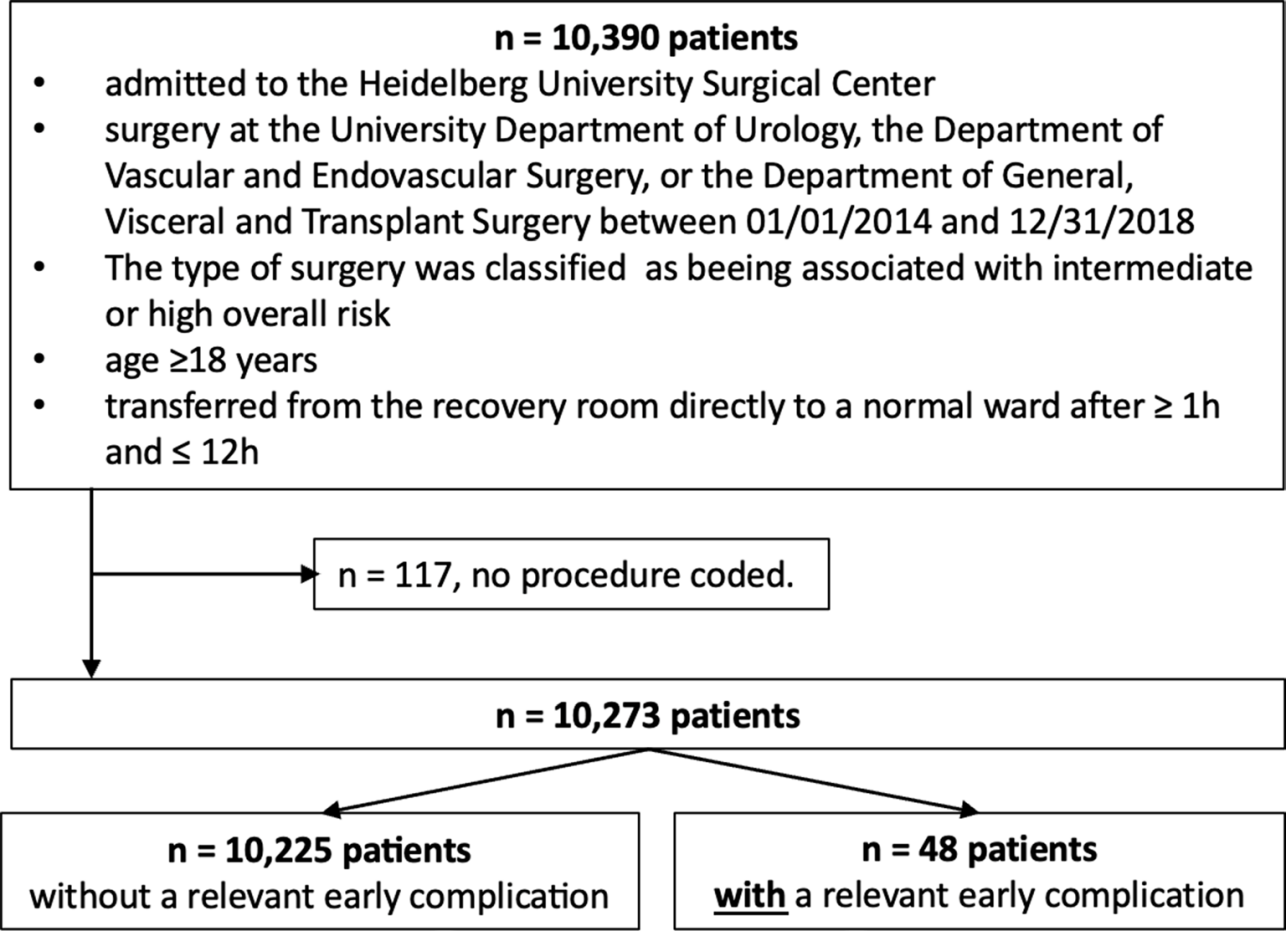
Flow chart detailing patient selection

During the observation 41,301 patients were treated in the PACU, about 80% of whom had undergone medium or major surgery; 10,273 patients met all inclusion criteria for this study. A total of 48 of these patients suffered an REC (prevalence = 0.47%; confidence interval [CI] = 0.4–0.6%). Figure 2 details the inclusion process, and *Online Source Table SI* provides insight into characteristics of patients admitted to the PACU during the study period 2014–2018.

### Study population

Approximately two thirds (66.9%) of patients in the study population underwent surgery performed by the Department of General, Visceral, and Transplant Surgery, 11.5% underwent surgery performed by the Department of Vascular and Endovascular Surgery, and 21.6% underwent surgery performed by the Department of Urology (*Online Source Table SI 2*). The types of surgical procedures performed, which have been classified by the operating department, are detailed in the *Online Source Tables SI 3–5*. Of the 48 patients with early complications, 36 (75%) received surgery from the Department of General, Visceral, and Transplant Surgery (prevalence = 0.59%, CI = 0.38–0.72%), 7 (14.6%) received surgery from the Department of Vascular and Endovascular Surgery (prevalence = 0.60%, CI = 0.29–1.22%), and 5 (10.4%) received surgery from the Department of Urology (prevalence = 0.23%, CI = 0.10–0.53) (*Online Source Table SI 2*). REC prevalence did not differ significantly between the surgical departments (*p* = 0.16). However, the prevalence of RECs was significantly higher in the group of patients who had undergone surgery in the “high” risk category (prevalence = 0.69%, CI = 0.46–1.06%) than in the group of patients who had undergone medium surgery (prevalence = 0.37%, CI = 0.26–0.54; *p* = 0.04) [13, 21].

### Patient characteristics

There were no relevant differences between patients with and without RECs with respect to age (*Online Source Table SI 6*), BMI, pre-existing comorbidities (based on the CCI), cardiovascular burden (RCRI; *Online Source Table S7*), or representation of ASA classes (Table 1). However, women were slightly overrepresented in the group of patients with REC. Furthermore, surgical procedures in the high-risk category were more often associated with RECs than were operations in the medium-risk category (Table 1).

**Table 1:** Patient characteristics

	Total *n* (%)		Without REC *n* (%)		With REC *n* (%)		*p*
	**10**,**273**		**10**,**225**	(99.5)	**48**	(0.5)	
Gender n (%)*							
Male	6,525	(63.5)	6,502	(63.6)	23	(47.9)	**0.025**
Female	3,748	(36.5)	3,723	(36.4)	25	(52.1)	
**Age** (years)*							
Mean (SD)	60.76	(14.0)	60.8	(14.0)	63.21	(13.6)	
Median (IQR)	63.00	(53 − 71)	63	(53–71)	64	(57–75)	0.17
RCRI* (Points)							
0, n (%)	8.091	(78.8)	8.054	(78.4)	37	(77.1)	
1, n (%)	1.715	(16.7)	1.709	(16.6)	6	(12.5)	
2, n (%)	376	(3.7)	373	(3.6)	3	(6.3)	
3, n (%)	81	(0.8)	79	(0.8)	2	(4.2)	
4, n (%)	10	(0.1)	10	(0.1)	0	(0.0)	0.07
Mean (SD)	0.3	(0.6)	0.3	(0.6)	0.4	(0.8)	
Median (IQR)	0.00	(0.0–0.0)	0.00	(0.0–0.0)	0.0	(0.0–0.0)	
**BMI** (kg/m^2^)							
Median (IQR)	25.6	(22.8–28.7)	25.6	(22.8–28.8)	25.1	(22.8–27.8)	0.82
Missing values n (%)	364	(3.5)	358	(3.5)	6	(12.5)	
CCI* (Points)							
Mean (SD)	2.9	(3.2)	2.9		3.21	(3.4)	0.28
Median (IQR)	2.00	(0.0–4.0)	2.00	(0.0–4.0)	2.00	(0.3–5.5)	
ASA classification *							
**I**	761	(7.4)	754	(7.4)	7	(14.6)	0.06
**II**	5.585	(54.4)	5.563	(54.4)	22	(45.8)	
**III**	3.818	(37.2)	3.801	(37.2)	17	(35.4)	
**IV**	103	(1.0)	101	(1.0)	2	(4.2)	
**V**	6	(0.1)	6	(0.1)	0	(0.0)	
Cardiovascular risk category of surgery*							
High n (%)	3.022	(29.4)	3.002	(29.4)	21	(43.8)	**0.04**
Medium n (%)	7.251	(70.6)	7.223	(70.6)	27	(56.3)	
Emergency status *							
n (%) requiring immediate surgery or surgery within 2 h	373	(3,6)	367	(3.6)	6	(1.6)	**0.001**

*—No missing values; ASA—American Society of Anesthesiologists classification; BMI—Body Mass Index; CCI—Chalson Comorbidity Index; RCRI—Revised Cardiac Risk Index; REC—relevant early complication; IQR—interquartile range (25th–75th percentile); SD—standard derivation

### Length of PACU stay

Regarding the PACU stay following the initial operation, there was no significant difference in median length of PACU between patients with and without RECs (350 min (IQR 255–405 min) vs. (285 min; IQR 210–360 min); *p* = 0.11). There were no notable differences in length of PACU stay between patients with different types of RECs.

### Recorded RECs

A total of 48 patients experienced an REC within the first 24 h of discharge from the PACU (prevalence 0.50%, CI: 0.40–0.60%). A total of 15 (31.3%) of these complications were postoperative bleeding (prevalence = 0.15%, CI = 0.09–0.24%), and seven cases (14.6%) were cardiovascular complications (prevalence = 0.07%, CI = 0.03–0.14%). In six cases, the REC was pulmonary artery embolism (PAE, prevalence = 0.06%, CI = 0.03–0.14%). In five patients REC was an ischemia of an extremity (prevalence = 0.05%, CI = 0.02–0.11%). In six patients (prevalence = 0.06%, CI = 0.03–0.14%) an abdominal problem (such as transaminase increase, or insufficiency of biliodigestive anastomosis) led to readmission to the PACU. In five cases the REC was a respiratory problem in (prevalence = 0.05%, CI = 0.02–0.11%), acute renal failure in two cases (prevalence = 0.02%, CI = 0.02–0.11%), a neurological problem in one case, and pain that was unmanageable on NWs in one case. Hospital mortality following RECs was 0.01% (CI = 0.001–0.055%; 1/10,273), as one patient died due to PAE following liver surgery. Surgical intervention was required in 18 cases due to RECs (37.5%), and in two additional cases bleeding was stopped by radiological intervention.

### Surgeries with RECs

Out of the 48 recorded RECs, 36 RECs (75.0%; prevalence = 0.59%, CI = 0.38–0.72%) occurred following visceral surgeries. These surgeries were 2 exploratory laparoscopic procedures, 2 partial small bowel resections, 5 partial colon resections, 3 open rectal resections, 10 liver surgeries, 13 pancreas surgeries, 2 exploratory laparotomies, and 2 unspecified surgeries. RECs occurred in seven vascular surgical procedures (14.6% of all RECs; prevalence following vascular surgery procedures = 0.60%, CI = 0.29–1.22%) as follows: postoperative ischemia occurred in three bypass procedures; shunt re-occlusion occurred once after shunt thrombectomy; re-ischemia occurred once following femoral endarterectomy; one postoperative hemorrhage occurred after placement of a bifurcation prosthesis of the abdominal aorta; and postoperative respiratory decompensation occurred once. Five RECs occurred following urological surgery (10.4% of all RECs; REC prevalence following urological procedures = 0.23%, CI = 0.10–0.53%), arising as follows: three RECs consisted of bleeding following Da Vinci robot-assisted prostatectomies, one REC was an orthostatic syncope in the context of the first post-surgical mobilization on the ward, and one REC was a hypertensive emergency that led to readmission to the PACU.

The *Online Source Tables SI 8–S10* provide detailed overviews of the RECs that occurred for each type of surgery. A high prevalence of a specific type of REC in a specific procedure was not observed. Due to the small numbers of cases within such subgroups, further statistical evaluation was not deemed to be appropriate.

### Patient characteristics grouped by type of REC

The characteristics of patients with and without RECs were compared separately for the most frequent REC types, as well as for anesthesiologically relevant RECs such as re-bleeding, cardiovascular complications, pulmonary artery embolism (PAE), and respiratory complications (Table 2).

**Table 2:** Characteristics of patients grouped by type of early complication

	Without REC *n* (%)		Postoperative bleeding *n* (%)			Cardiovascular *n* (%)			PAE *n* (%)			Respiratory *n* (%)		
	10,225		15 (31.3)		p	7	(14.6)	p	6	(12.5)	p	5	(10.4)	p
**Prevalence (%)**			0.15 (CI = 0.09–0.24)			0.07 (CI = 0.03–0.14)			0.06 (CI = 0.03–0.14)			0.05 (CI = 0.02–0.11)		
Gender*														
Male n (%)	6,502	(63.6)	8	(53.3)	0.7	4	(57.1)	0.7	3	(50)	0.5	3	60	0.1
Female n (%)	3,723	(36.4)	7	(46.7)		3	(42.9)		3	(50)		2	40	
Age*														
Median (IQR)	63	(53–71)	64	(56.5–67.0)	0.5	77	(72–78.5)	**0.002**	64	(58.5–71)	0.3	55	(52–75)	0.1
RCRI* (points)														
0, n (%)	8,054	(78.4)	12	(80.0)	0.6	5	(71.4)	0.4	6	(100)		3	60.0	0.1
1, n (%)	1,709	(16.6)	2	(13.3)		1	(14.3)					1	20.0	
2, n (%)	373	(3.6)	1	(6.7)			(0.0)						0.0	
3, n (%)	79	(0.8)	0	(0.0)		1	(14.3)					1	20.0	
4, n (%)	10	(0.1)	0	(0.0)			(0.0)							
**BMI** (kg/m^2^)														
Median (IQR)	25.6	(22.8–28.8)	25.6	(23.7–28.1)	0.9	25.1	(24.7–25.3)	0.8	28.3	(26.3–30.8)	0.3	25.9	(22.8–28.9)	0.1
Missing values n (%)	358	(3.5)	1	(6.7)		0	(0)		0	(0)		1	(0.2)	
CCI* (points)														
Median (IQR)	2	(0–4)	2	(0–3.5)	0.8	6	(2–8)	0.1	0.5	(0–1.8)	0.3	3	(2–9)	0.1
ASA class* n (%)														
**I**	754	(7.4)	2	(13.3)	0.8	0	(0.0)	0.1	1	(16.7)	0.1	1	(20.0)	0.3
**II**	5,563	(54.4)	9	(60.0)		3	(42.9)		5	(83.3)		3	(60.0)	
**III**	3,801	(37.2)	4	(26.7)		3	(42.9)		0	(0.0)		1	(20.0)	
**IV**	101	(1.0)	0	(0.0)		1	(14.3)		0	(0.0)		0	(0.0)	
**V**	6	(0.1)	0	(0.0)		0	(0.0)		0	(0.0)		0	(0.0)	
Cardiovascular surgical risk*														
High n (%)	3,002	(29.4)	3	(20)	0.4	3	(42.9)	0.4	4	(66.7)	**0.04**	0	0	0.2
Medium n (%)	7,223	(70.6)	12	(80)		4	(57.1)		2	(33.3)		5	100	

*—No missing values; p— p-values from comparison between the respective subgroup and the group of patients without REC; ASA—American Society of Anesthesiologists; CI—Confidence interval; CCI—Charlson Comorbidity Index; IQR—interquartile range = 25th–75th percentile; PAE—pulmonary artery embolism; RCRI—Revised Cardiac Risk Index; SD—standard derivation

The prevalence of postoperative bleeding was 0.15% (CI = 0.09–0.24%). A total of five cardiovascular RECs were observed (prevalence = 0.07%; CI = 0.03–0.14%): there were two cases of tachyarrhythmia absoluta, and in both cases permanent atrial fibrillation was a known pre-existing condition; two syncopes occurred following initial mobilization; and one REC was a hypertensive emergency. Six cases of PAE were observed (prevalence = 0.06%, CI = 0.03–0.14%). PAE occurred once after extensive adhesiolysis of the bowels, once after left hepatic resection, and four times after pancreatic surgery; one case of PAE was fatal.

Patients with cardiovascular RECs were on average 13.7 years older than patients without cardiovascular RECs (mean 60.7 vs. 74.4 years, respectively; *p* = 0.002; Table 2). There were no other notable differences in risk profiles (gender, RCRI, BMI, CCI, or ASA status) between patients with and without re-bleeding, cardiovascular complications, or PEA as RECs (Table 2).

The prevalence of early respiratory complications was 0.05% (CI = 0.02–0.11%). Three patients were readmitted to the PACU after open bowel surgery due to respiratory decompensation. This occurred once after exploratory laparotomy and once after pancreatic surgery. These patients did not differ from those without early respiratory complications in terms of sex, age, RCRI, BMI, CCI, ASA status, or cardiovascular risk status of the surgical procedure. The prevalence of pre-existing renal dysfunction (preoperative serum creatinine ≥ 2) was 20.0% (1/5) in patients with respiratory RECs compared to 8.3% (846 of 10,225) in patients without respiratory REC (*p* = 0.35). Three out of five patients (60.0%) with respiratory RECs and 2,266 out of 10,225 of patients (22.0%) without respiratory RECs (22.0%) were active smokers; this difference was not significant (*p* = 0.08) due to the small number of cases in the group with RECs. However, respiratory RECs were associated with pre-existing obstructive lung disease (60.0% (3 of 5) in patients with RECs vs. 7.7% (783 of 10,225) in patients without RECs; *p* < 0.01).

### PACU admission during the day versus night and emergency status

In order to detect a possible dependence of the length of PACU stay on the time at PACU admission—specifically daytime versus nighttime admission—length of PACU stay was compared between patients who were admitted to the PACU during the day (7:00 am–6:55 pm) and the night (7:00 pm–6:55 am); no significant differences were detected (Fig. SI 1). However, patients admitted to the PACU at night (7:00 pm–6:55 am) were more likely to have RECs than patients admitted to the PACU in daytime (REC prevalence 1.20% (15/1,258) vs. 0.37% (33/9,015); *p* = 0.0001). Likewise, patients who were transferred from the PACU to a NW during the night (7 pm–6:55 am) were more likely to have RECs (REC prevalence 0.63% (34/5,118) vs. 0.27% (14/5,155); *p* = 0.004).

Notably, REC prevalence was not higher in emergency patients when they were admitted at night to the PACU (REC prevalence in patients with emergency status admitted to the PACU at daytime (7:00 am–6:55 pm) 3/97 vs. 3/241 at night (7:00 pm–6:55 am); *p* = 0.2) or when they were transferred at night to the NW (REC prevalence in patients with emergency status transferred from the PACU to the NW at daytime 5/220 vs. 1/162 at nighttime; *p* = 0.2). REC prevalence was significantly lower in the subgroup of patients undergoing elective surgery than in emergency patients (REC prevalence in elective surgery patients 0.42% (42/9888) vs. 1.61% (6/367) in patients with emergency status; *p* = 0.001). In the subgroup of patients who were [1] undergoing elective surgery [2], being admitted to the PACU between 7:00 am and 6:55 pm, and [3] being transferred (after 1–12 h) from the PACU to the NW between 7:00 am and 6:55 pm, REC prevalence was 0.23% (12/5254).

#### Evaluation of the relevance of RECs in pancreatic surgery

The secondary objective of the present work was to determine the prevalence of RECs in the high-risk subgroup of patients who underwent partial duodenopancreatectomy (known as the Whipple procedure). During the study period, a total of 740 Whipple operations were performed, after which patients were monitored in the PACU for 1–12 h before transfer to a NW; these included 407 classical Whipple surgeries and 333 pylorus-preserving (pp) Whipple surgeries. Of these 740 patients, 8 patients (prevalence = 1.10%, CI = 0.55–2.12%) suffered an REC (Table 3). All 740 Whipple procedures were elective surgery, and most of the patients undergoing a Whipple procedure were admitted to the PACU during the day (632/732, 86.3%); no RECs occurred in Whipple patients who were admitted at night to the PACU. Although it should be noted that most patients who had undergone pancreatic surgery were transferred from the PACU to the NWs during the night, this was not associated with an increased prevalence of REC (REC prevalence in patients transferred to normal ward during the night 1.3% (1/77) vs. 1.06% (7/663) in patients transferred between at day time; *p* = 0.85). However, a closer look at the transfer times from the PACU to the NW revealed that all patients with REC were transferred to the NW particularly late, specifically, between 0:00 and 4:00 am (*Fig. SI 2*).

**Table 3:** Classification of underlying disease, pancreatic surgery, and complication

Underlying disease	Surgery	Complication
IPMN	ppWhipple surgery	PAE
IPMN	ppWhipple surgery	BDA insufficiency
Duodenal carcinoma	Whipple surgery	BDA insufficiency
Pancreatic head carcinoma	ppWhipple surgery	PAE
Pancreatic head carcinoma	Whipple surgery	PAE
Pancreatic head carcinoma	Whipple surgery	Syncope
Pancreatic head carcinoma	Whipple surgery	Bleeding
Pancreatic head carcinoma	ppWhipple surgery	Bleeding

BDA—biliodigestive anastomosis; IPMN—intraductal papillary mucinous neoplasia; PAE—pulmonary artery embolism; ppWhipple—Whipple pylorus-preserving surgery; Whipple surgery—partial pancreaticoduodenectomy

Three of the eight RECs in this sub cohort were PAEs (prevalence = 0.40%, CI = 0.21–1.39%), two cases consisted of re-bleeding (prevalence = 0.30%, CI = 0.05–0.99) and two cases of insufficiencies of the biliodigestive anastomosis (prevalence = 0.30, CI = 0.05–0.99). In both cases, the insufficiencies of the biliodigestive anastomosis were detected by routine checks of the bilirubin concentration in abdominal drains. In both cases patients underwent another surgery. One case was an orthostatic syncope during initial postoperative mobilization (prevalence = 0.10, CI = 0.01–0.77). The median duration of Whipple surgery was slightly longer in patients with than without a subsequent REC (315 min; IQR: 260–365 min compared to 393 min IQR: 253–293 min, respectively; *p* = 0.84); likewise, there was no relevant difference in the median length of stay in the PACU (patients without REC: 355 min; IQR: 281–420 min vs. patients with RECs: 353 min; IQR: 280–538 min; *p* = 0.52). Similarly, no differences between patients with and without RECs were identified regarding age structure, BMI, representation of ASA classes, preoperative RCRI, the type of perioperative analgesia used (epidural catheter vs. opioids) or CCI (Table 4). Of note, among Whipple procedure patients, those with a subsequent REC had a longer length of hospital stay (LOS) than those without an REC (“with REC” median LOS: 16 days (IQR 14–17) vs. “without REC” median LOS: 12 days (IQR 10–17), *p* = 0.021). All 740 patients following Whipple surgery and being transferred to a NW after a PACU stay of (≥ 1 h and) ≤ 12 h survived until discharge from the hospital.

**Table 4:** Characteristics of patients who underwent Whipple surgery

	Whipple surgery without REC *n* (%)		Whipple surgery with REC *n* (%)		*p*	Whipple surgery with partial gastric resection *n* (%)		ppWhipple surgery *n* (%)		
	732		8	1.1		407	55.0	333	45.0	
Gender* **n (%)**										
Male	396	(54.1)	2	(25.0)	0.15	226	(55.5)	172	(51.7)	
Female	336	(45.9)	6	(75.0)		181	(44.5)	161	(48.3)	
**Age** (years)*										
Median (IQR)	64.0	(54.0–72.0)	67.5	(57.0–78.0)	0.4	64.00	(54.0–73.0)	64.00	(53.5–72.0)	
Revised Cardiac Risk Index* (RCRI) (points)										
0. n (%)	608	(83.1)	7	(87.5)	NA	4	(100.0)	3	(75.0)	
1, n (%)	109	(14.9)	1	(12.5)				1	(25.0)	
2, n (%)	12	(1.6)								
3, n (%)	3	(0.4)								
4, n (%)	0	(0.0)								
Hyper-tension*										
n (%)	342	(46.7)	4	50.0	0.9	195	(47.9)	147	(44.1)	
**COPD**										
n (%)	45	(6.1)	1	12.5	0.5	26	(6.4)	19	(5.7)	
Charlson Comorbidity Index* (points)										
Median (IQR)	2.00	(0.0–8.0)	5.00	(1.5–8.3)	0.6	3.0	(1.0–8.0)	2.00	(0.0–8.0)	
ASA class* **n (%)**										
**I**	48	(6.6)	1	(12.5)	NA	27	(6.6)	22	(6.6)	
**II**	461	(63.0)	5	(62.5)		243	(59.7)	223	(67.0)	
**III**	221	(30.2)	2	(25.0)		136	(33.4)	87	(26.1)	
**IV**	1	(0.1)	0	(0.0)		1	(0.3)	0	(0.0)	
**V**	1	(0.1)	0	(0.0)		0	(0.0)	1	(0.3)	
Analgesia*										
**PDA**	454	(61.3)	4	(50.0)	0.5	202	(49.6)	256	(76.9)	

*—No missing values; ASA American Society of Anesthesiologists; CCI—Charlson Comorbidity Index; COPD—chronic obstructive pulmonary disease; IQR—interquartile range = 25th–75th percentiles; NA—not applicable; ppWhipple—pylorus-preserving Whipple surgery; RCRI—Revised Cardiac Risk Index; Whipple surgery—partial pancreaticoduodenectomy

## Discussion

The necessary length of stay in a PACU of orientated (to situation, time, place and person) and respiratory and cardio-circulatory stable patients depends essentially on the probability of a surgical complication. Such patients do not require organ support and therefore do not require intensive care medicine in proper sense. The term cardio-circulatory stable also implies that fluid management in these patients is feasible without the special facilities of an ICU. The indication for hospitalization of such patients in an ICU therefore only arises from the stochastic risk of a complication that occurs so quickly or unnoticed that it cannot be recognized in the early stages by either the patient or the nursing staff on the NWs and the patient is put at risk by the delayed start of treatment. Our retrospective study shows that it is possible, through individual case-by-case decisions, to select a subset of patients from the large group of patients after medium or major operations who, after being monitored for 1–12 h in the PACU, have a low risk of relevant complications within the first 24 h on a NW.

We are aware that this 12-hour limit is arbitrary to a certain extent. The idea behind this study was to find out whether patients who are stable after elective surgery, which is usually performed in the morning, can be safely transferred from the PACU to a NW on the same day. In order to also incorporate patients who were operated on in the late afternoon or who were treated as emergencies at night, the 12-hour rule appeared to be a reasonable surrogate for time-limited monitoring in the PACU. For similarly pragmatic reasons, the 24-hour duration of observation on the NW was chosen—the aim was to record complications that would have occurred in the ICU if the patients had remained in the ICU for one day longer.

Therefore, with regard to its objective, our work significantly differs from other studies that analyzed more global outcome parameters. One the one hand, there are already numerous studies that attempt to estimate a patient’s prognosis based on patient characteristics or surgical parameters and to evaluate the performance of fast-track programs [27–32]. However, in contrast to our work, the outcome parameters used in other studies are, for example, the length of hospitalization, the possibility of early discharge from hospital, or the discharge destination after hospitalization [27–32]. Enhanced Recovery After Surgery (ERAS) protocols, on the other hand, focus mainly on perioperative pain management and perioperative nutrition [33]. However, the retrospective analysis presented here is not designed to draw conclusions about the possible effects of a prolonged postoperative ICU stay on the long-term outcome of patients after medium and major surgery. The statements we can make are as follows: (I) Through the case-by-case assessment of a surgeon in addition to an anesthesiologist, it was possible to select patients who had a low probability of requiring ICU treatment again in the following 24 h. (II) The hospital mortality rate of the patients selected in this way was low (at 0.01%) and significantly below the expected mortality rate in the patients who had undergone medium (30-day mortality = 0.7–5%) and major operations (30-day mortality > 5%) [13, 34]. (III) Emergency cases and patients who were admitted to the PACU at night or transferred from the PACU to the NW had an increased REC risk. (IV) The subgroup analysis of patients who had undergone pancreaticoduodenectomy showed that even in this group of elective complex operations in the high risk category, medical experience can be used to select patients who have a low risk of RECs. (V) Although, Patients after pancreaticoduodenectomy who had a REC had a longer median hospital length of stay than patients after pancreaticoduodenectomy without REC, none of the patients following pancreaticoduodenectomy included in our study died during their hospital stay. Even this prolonged length of hospitalization was still significantly below the national average for length of hospitalization after pancreatic surgery (25 days) [35].

In order to gain further insight into the usefulness or necessity of ICU monitoring in such a medically preselected group, we took a closer qualitative look at the observed RECs; quantitative analyses did not seem appropriate in view of the low number of complications. As the overall study population includes a wide range of operations whose procedure-specific risks can only be compared to a limited extent, such qualitative analyses would also only be of limited value. The RECs following duodenopancreatectomies were therefore analyzed as an example. This subgroup only included patients who underwent planned (non-emergency) surgery during the daytime, and most of them were transferred from the PACU to the NW in the evening. However, it is noticeable that the patients with RECs were transferred particularly late, so that the transfer time factor should not be completely ruled out as a contributory causal factor. The early complications of hemorrhage and PAE in the subgroup of patients who had undergone partial pancreaticoduodenectomy are discussed here as examples in more detail.

The most frequently observed RECs after pancreaticoduodenectomy were PAEs; this is surprising in itself as one would expect PAE to occur with a few days latency after surgery as a result of deep vein thrombosis. For example, Hope et al. reported a median latency from surgery to embolic onset of three days in young patients (under 40 years of age) and 11 days in patients between 40 and 60 years old [36]. Other studies have reported median latencies of 8 to 34 days between surgery and diagnosis of PAE [37, 38]. Considering these findings, it is striking how frequently we observed PAEs as RECs. The authors of an autopsy study investigating the significance of immediate postoperative fatal PAEs concluded that, in such cases of very early PAE, thrombosis-promoting factors such as fever (as a surrogate for systemic inflammation) or prolonged immobility were already present at or before the time of surgery [39].

On the one hand, the subgroup of patients undergoing surgery for a pancreatic tumor has an increased risk of PAE for this reason alone [40, 41]. On the other hand, thrombosis of the iliac veins and vena cava should be detected in the preoperative-planning imaging. However, there is a certain latency between planning imaging and surgery. In addition, the leg veins are usually not part of this imaging, so it may well be that patients go into surgery with an unrecognized thrombosis. Overall, it must be emphasized that PAE remains a rarity even in such high-risk patients [41]. In contrast, the prevalence of PAE in hospital is reported to be around 0.4% in patients undergoing hip or knee replacement [42, 43]; these patients are transferred to a NW on the day of surgery in most hospitals and are increasingly being operated on in a fast-track procedure or even as outpatients. Thus, postoperative PAE is a complication that occurs in most cases in NWs, in a rehabilitation center, or even at home. Therefore, it does not seem reasonable to monitor clinically stable patients in an ICU for a longer period of time after moderate and major surgery simply because PAE is a concern. It also seems unlikely that a different timing of transfer from the PACU to the ICU would have prevented the occurrence of PAE, although we acknowledge that, due to the retrospective nature of our work, we have no clear evidence in favor of this hypothesis.

In this study, the prevalence of postoperative hemorrhage within the first 24 h after discharge from the PACU was 0.3% in patients undergoing pancreaticoduodenectomy. In the literature, a distinction is made between “early” bleeding complications, which occur within the first 24 h after completion of the surgical procedure, and “late” bleeding complications, which only become symptomatic after the first 24 postoperative hours [44]. The reason for the delineation at 24 h is that bleeding complications during this time are most likely to be caused by a technical error, such as by inadequate haemostasias, or by coagulopathy [44]. In contrast, “late” bleeding complications usually occur days to weeks after the operation [44]; these are usually caused by septic erosion of abdominal vessels, for example as a result of a pancreatic fistula; ulcers at the anastomoses; or the development of an arterial pseudoaneurysm [44]. Early postoperative hemorrhage is generally less severe than late postoperative hemorrhage [45–47]. When analyzing the bleeding complications we observed, it should be noted that we only counted bleeding complications that occurred in patients who had no evidence of ongoing blood loss or coagulopathy after the monitoring period in the PACU, as only these patients were transferred to a NW. Cases of bleeding that occurred during the monitoring period in the PACU were not included in our analyses. Thus, our work specifically captures those bleeding complications that occurred in the first 24 h after transfer from the PACU to the NW that could not be monitored and managed on a normal ward. Of course, one could theoretically imagine that we would have missed a hemorrhage that occurs after 23 h, is first noticed after 24 h and leads to readmission to the OR/PACU only after 25 h. However, this is a natural limitation of any study. For example, the commonly used 28-day mortality does not include deaths on day 29. Considering that re-bleeding in the first 24 h is most likely due to technical errors during surgery, such as insufficient hemostasis or incomplete vascular ligation, it is very likely that these cases of bleeding will become symptomatic in the first postoperative hours in the PACU (through a drop in hemoglobin level, pain, or bleeding through any drains present). In particular, this applies to fulminant bleeding from arteries or large veins. Bleeding complications that become symptomatic at a later stage are likely to be of lower volume and therefore have slower kinetics, giving physicians more time to react before the hemorrhage becomes life threatening [44]. It is therefore less likely that a critical situation such as postoperative bleeding in a preselected patient subgroup such as we have described will become a danger to the patient. This is especially true as an important transfer criterion is the patient’s mental state. Patients with a Glasgow Coma Scale of 15 can actively call for help themselves when symptoms such as pain or discomfort occur.

The question remains as to whether prolonged postoperative monitoring and treatment can prevent the development of complications. In an observational study, Ludbrook et al. investigated whether patients at intermediate overall risk (defined as a predicted 30-day mortality of 0.7–5%) could benefit from an extended monitoring period [34]. Depending on availability, patients were either assigned to a dedicated monitoring area (ARRC; *n* = 452)) with capacity for invasive monitoring and vasoactive infusions on the first postoperative night, or they were transferred to the NW after the usual PACU stay (*n* = 419) [34]. Although medical emergencies were less frequent between days two and nine in the group receiving ARRC care (9 [2.6%] vs. 22 [6.3%]; *p* = 0.03), there were no differences in terms of length of hospital stay, hospital readmissions, emergency department visits, or mortality [34]. Thus, Ludbrook et al. also succeeded in managing all complications arising on NWs by providing an adequate emergency concept in such a way that the impact on the patients was low. It is noteworthy that the study by Ludbrook et al. included unselected patients after intermediate surgery rather than clinically preselected patients, which was the case in our study. Of course, one would hope that emergency interventions would not be necessary at all. At the same time, it makes no sense, either for the individual patient or for society as a whole, to monitor a stable patient who does not have a significantly increased surgical risk in an intensive care unit for days and weeks [48–50]. For example, intensive care stays are suspected of contributing causally to the development of delirium [51–53], which in turn is associated with longer hospital stays and increased overall morbidity and mortality [51, 52].

The main strength of our work is the large number of cases analyzed, including both the overall number of patients and the subgroup of well-characterized patients who had undergone pancreaticoduodenectomies. However, an inherent limitation of our work is its retrospective observational nature. Consequently, the results presented here are, by definition, only hypothesis-generating observations that would need to be tested in a prospective, preferably randomized, controlled trial. In this context, it would also be interesting to record longer follow-up periods to enable the recording of long-term effects of intensive care treatment or the lack of intensive care treatment. However, in view of the figures presented here, such confirmation is probably not possible in practice. As the prevalence is low and the complications are very diverse, extremely high case numbers would be required to measure effects on the occurrence of early complications. Instead, further research is essential to (1) develop tools to identify patients suited to a shortened stay in the recovery ward and (2) find patient-centered outcome parameters that can reliably measure the performance of these tools. Another limitation of our work is the fact that the HIS (IS-H-med™) is not designed to capture early complications, as complications are not labeled or listed as such. We therefore had to take an indirect approach by selecting patients based on information on inter-ward transfers and the bed occupancy lists of emergency departments and intensive care units. Patients admitted to the operating theater were listed in the PACU, and they were selected for inclusion in this study if they were transferred to an ICU after their initial stay in the PACU and then transferred back to an ICU after up to 24 h; this can lead to false-negative and false-positive selections. To minimize the false-positive rate, all selected cases were individually reviewed by a physician and, for example, scheduled second-look surgeries were excluded. False-negative cases are less likely because a patient is admitted to the ICU with a complication requiring intensive care monitoring or therapy. Deaths on NWs within the 24 h observation period did not occur but would also have been counted as a complication. Another weakness inherent in our study, as in most large secondary evaluations, is that in the large group of REC-negative patients, medical data were not cross-checked in all cases due to the extensive time and effort that would have been involved. However, this was performed in the smaller group of patients with REC. It should also be noted that, overall, the data is subject to multiple controls, as coded principal diagnoses, procedures, and secondary diagnoses are part of billing in addition to forming the basis of surgical planning. They are not only checked by internal coding officers, but are also randomly checked by the independent medical service of the health insurance provider in order to avoid incorrect billing. The anesthesiology data is collected by an anesthesiologist, cross-checked preoperatively by a senior physician, and checked again for completeness before being entered into the HIS. Furthermore, we are aware that the selection of patients who were transferred to an ICU after a few hours of monitoring can be objective only to a limited degree, as it was based on the informed but subjective judgment of two experienced physicians. Although, from a scientific point of view, a standardized, purely score-based procedure (as would be required in a prospective, randomized study) would be preferable, we were only able to describe the procedure used during the observation period. However, it must be recognized that the physicians involved were clearly able to target patients with a low risk of complications within the first 24 h on a NW. This emphasizes the fact that multifactorial, high-quality decisions can be made during a medical examination, even if these can only be represented to a limited extent by prediction scores.

The data presented here were obtained in a highly specialized environment. Therefore, they can only be adapted to less specialized hospitals with caution; however, such adaptation is not impossible. From the authors’ point of view, there are three critical influencing factors. Firstly, the objective rate of surgical complications. Secondly, the surgeon’s self-assessment, as this is much more difficult to objectify using scoring systems than the anaesthesiological transfer decision. Thirdly, a functioning emergency system, with the possibility for the wards to call in both surgeons and intensive care physicians quickly and easily for support; only this infrastructure can effectively ensure that potential patient endangerment in the event of incorrect decisions can be minimized. Special care should be taken when adapting such a procedure to patients of 90 years of age and above and to ASA 5 patients—such patients were included in our study population, but only as a small proportion. Therefore, further work is needed to confirm the safety of the described approach in this particular group, which is beyond the focus of the present work.

## Conclusion

We found that clinically stable patients that were monitored in the PACU for only up to 12 h after medium and major surgery but were cleared both by a surgeon and an anesthesiologist to be transferred to a normal ward, had a low prevalence of needing intensive care treatment within the first 24 h following the transfer. This prevalence was particularly low in patients who underwent elective surgery and were monitored in the PACU during daytime. Further research is needed to predict more clearly, and with better objectifiable criteria, whether a patient will benefit from postoperative admission to an ICU or whether monitoring for a few hours in the PACU is sufficient.

## Electronic supplementary material

Below is the link to the electronic supplementary material.

Supplementary Material 1

## Data Availability

The datasets generated and/or analysed during the current study are available from the corresponding author, Dr. Thomas Schmoch (thomas.schmoch@uk-essen.de) on reasonable request.

## References

[1] Deutsche Gesellschaft für Anästhesiologie und Intensivmedizin (DGAI) (2009) Berufsverband Deutscher Anästhesisten (BDA). Überwachung Nach Anästhesieverfahren - Empfehlung Der Deutschen Gesellschaft für Anästhesiologie und Intensivmedizin Und Des Berufsverbandes Deutscher Anästhesisten. Anästh Intensivmed 7(50):S486–S489

[2] Vimlati L, Gilsanz F, Goldik Z (2009) Quality and safety guidelines of postanaesthesia care: Working Party on Post Anaesthesia Care (approved by the European Board and Section of Anaesthesiology, Union Européenne Des Médecins Spécialistes). Eur J Anaesthesiol September 26(9):715–721 10.1097/EJA.0b013e32832bb68f 19390443

[3] De Pietri L, Montalti R, Begliomini B (März 2014) Anaesthetic perioperative management of patients with pancreatic cancer. World J Gastroenterol 20(9):2304–23207 10.3748/wjg.v20.i9.2304 PMC3942834 24605028

[4] Whitaker Chair DK, Booth H, Clyburn P, Harrop-Griffiths W, Hosie H, Kilvington B (2013) u. a. Immediate post-anaesthesia recovery 2013: Association of anaesthetists of Great Britain and Ireland. Anaesth März 68(3):288–297 10.1111/anae.12146 23384257

[5] Ghaffar S, Pearse RM, Gillies MA (2017) ICU admission after surgery: who benefits? Curr Opin Crit Care 1 Oktober 23(5):424–429 10.1097/MCC.0000000000000448 28777159

[6] Ohbe H, Matsui H, Kumazawa R, Yasunaga H (2022) Postoperative ICU admission following major elective surgery: a nationwide inpatient database study. Eur J Anaesthesiol | EJA Mai 39(5):436 34636358 10.1097/EJA.0000000000001612

[7] The International Surgical Outcomes Study group (2016) Global patient outcomes after elective surgery: prospective cohort study in 27 low-, middle- and high-income countries. BJA: Br J Anaesth 1 November 117(5):601–609 10.1093/bja/aew316 PMC5091334 27799174

[8] Pearse RM, Moreno RP, Bauer P, Pelosi P, Metnitz P, Spies C (2012) u. a. mortality after surgery in Europe: a 7 day cohort study. Lancet 22 September 380(9847):1059–1065 10.1016/S0140-6736(12)61148-9 PMC3493988 22998715

[9] Pearse RM, Holt PJE, Grocott MPW (2011) Managing perioperative risk in patients undergoing elective non-cardiac surgery. BMJ 5 Oktober 343:d5759 10.1136/bmj.d5759 21976704

[10] Filson K, Atherholt C, Simoes M, DiPalma M, John S, Reynolds R (Oktober 2018) u. a. post-operative vital signs: how often is too often? JCO. 20. 36(30suppl):210–210

[11] Zeitz K, McCutcheon H (2006) Observations and vital signs: ritual or vital for the monitoring of postoperative patients? Appl Nurs Res 1 November 19(4):204–211 10.1016/j.apnr.2005.09.005 17098158

[12] Kahan BC, Koulenti D, Arvaniti K, Beavis V, Campbell D, Chan M (2017) u. a. critical care admission following elective surgery was not associated with survival benefit: prospective analysis of data from 27 countries. Intensive Care Med 1 Juli 43(7):971–979 10.1007/s00134-016-4633-8 28439646

[13] Kristensen SD, Knuuti J, Saraste A, Anker S, Bøtker HE, Hert SD (2014) u. a. 2014 ESC/ESA guidelines on non-cardiac surgery: cardiovascular assessment and management: the Joint Task Force on non-cardiac surgery: cardiovascular assessment and management of the European Society of Cardiology (ESC) and the European Society of Anaesthesiology (ESA). Eur Heart J 14 September 35(35):2383–2431 10.1093/eurheartj/ehu282 25086026

[14] Aldrete JA (1995) The post-anesthesia recovery score revisited. J Clin Anesth 1 Februar 7(1):89–91 10.1016/0952-8180(94)00001-k 7772368

[15] Aldrete JA, Kroulik D (1970) A postanesthetic recovery score. Anesth Analg Dezember 49(6):924–934 5534693

[16] Gell G, Schmücker P, Pedevilla M, Leitner H, Naumann J, Fuchs H (2003) u. a. SAP and partners: IS-H™ and IS-H*MED™. Methods Inf Med Februar 42(1):16–24 12695792

[17] Auhuber T (2019) OPS 2020 Systematisches Verzeichnis: Operationen- und Prozedurenschlüssel; Internationale Klassifikation der Prozeduren in der Medizin. 1. Aufl. Köln: Deutscher Ärzteverlag; 853 S

[18] World Health Organization (2009) ICD-10 International Classification of Mortality and Morbidity Statistics - Tenth Revision [Internet]. [zitiert 29. Oktober 2009]. Verfügbar unter: http://www.dimdi.de/static/de/klassi/diagnosen/icd10/htmlgm2010/block-c69-c72.htm

[19] Doyle DJ, Goyal A, Garmon EH American Society of Anesthesiologists Classification. In: StatPearls [Internet]. Treasure Island (FL): StatPearls Publishing; 2022 [zitiert 1. Juni 2022]. Verfügbar unter: http://www.ncbi.nlm.nih.gov/books/NBK441940/

[20] Irlbeck T, Zwißler B, Bauer A (2017) [ASA classification: transition in the course of time and depiction in the literature]. Anaesthesist Januar 66(1):5–10 10.1007/s00101-016-0246-4 27995282

[21] Glance LG, Lustik SJ, Hannan EL, Osler TM, Mukamel DB, Qian F (2012) u. a. The Surgical Mortality Probability Model: derivation and validation of a simple risk prediction rule for noncardiac surgery. Ann Surg April 255(4):696–702 10.1097/SLA.0b013e31824b45af 22418007

[22] Landesärztekammer Baden-Württemberg. Berufsordnung der Landesärztekammer Baden-Württemberg [Internet] (2020) [zitiert 3. Juni 2022]. Verfügbar unter: https://www.aerztekammer-bw.de/10aerzte/40merkblaetter/20recht/05kammerrecht/bo.pdf

[23] The World Medical Association (WMA) Declaration of Helsinki – Ethical Principles for Medical Research Involving Human Subjects [Internet]. 2018 [zitiert 15. August 2019]. Verfügbar unter: https://www.wma.net/policies-post/wma-declaration-of-helsinki-ethical-principles-for-medical-research-involving-human-subjects/

[24] Brown LD, Cai TT, DasGupta A (2001) Interval estimation for a binomial proportion. Stat Sci Mai 16(2):101–133

[25] GraphPad Prism. GraphPad Prism 9 Statistics Guide - Three methods for computing the CI of a proportion [Internet]. [zitiert 8. September 2022]. Verfügbar unter: https://www.graphpad.com/guides/prism/latest/statistics/stat_three_methods_for_computing_th.htm

[26] Wilson EB (1927) Probable inference, the Law of Succession, and statistical inference. J Am Stat Association 1 Juni 22(158):209–212

[27] Walczak S, Velanovich V (2022) Predicting Elective Surgical patient outcome destination based on the Preoperative modified Frailty Index and Laboratory values. J Surg Res 1 Juli 275:341–351 10.1016/j.jss.2022.02.029 35339003

[28] Mahvi DA, Pak LM, Bose SK, Urman RD, Gold JS, Whang EE (2019) Fast-track pancreaticoduodenectomy: Factors Associated with early discharge. World J Surg Mai 43(5):1332–1341 10.1007/s00268-019-04916-0 30680502

[29] Mahvi DA, Pak LM, Urman RD, Gold JS, Whang EE (2019) Discharge destination following pancreaticoduodenectomy: a NSQIP analysis of predictive factors and post-discharge outcomes. Am J Surg 1 August 218(2):342–348 10.1016/j.amjsurg.2018.11.043 30553461

[30] Mahvi DA, Pak LM, Fields AC, Urman RD, Gold JS, Whang EE (2019) Prediction of Discharge Destination following major Hepatectomy. HPB 1 November 21(11):1462–1469 10.1016/j.hpb.2019.03.354 30956164

[31] Paredes AZ, Hyer JM, Tsilimigras DI, Bagante F, Beal EW, Merath K (2019) u. a. predictors and outcomes of nonroutine discharge after hepatopancreatic surgery. Surg 1 Juni 165(6):1128–1135 10.1016/j.surg.2019.02.020 30981416

[32] Kobayashi S, Ooshima R, Koizumi S, Katayama M, Sakurai J, Watanabe T (2014) u. a. Perioperative Care with fast-track management in patients undergoing pancreaticoduodenectomy. World J Surg 38(9):1 10.1007/s00268-014-2548-5 24692004

[33] Koerner AS, Thomas AS, Chabot JA, Kluger MD, Sugahara KN, Schrope BA (2023) Associations between patient characteristics and Whipple Procedure outcomes before and after implementation of an enhanced recovery after surgery protocol. J Gastrointest Surg 1 September 27(9):1855–1866 10.1007/s11605-023-05693-x 37165160

[34] Ludbrook G, Grocott MPW, Heyman K, Clarke-Errey S, Royse C, Sleigh J (2023) u. a. outcomes of postoperative overnight high-acuity care in medium-risk patients undergoing elective and unplanned noncardiac surgery. JAMA Surg 1 Juli 158(7):701–708 10.1001/jamasurg.2023.1035 PMC10157507 37133876

[35] Baum P, Diers J, Lichthardt S, Kastner C, Schlegel N, Germer CT (2019) Sterblichkeit Und Komplikationen Nach Viszeralchirurgischen Operationen. Deutsches Ärzteblatt 1 November 44(116):739–746 10.3238/arztebl.2019.0739 PMC6912125 31774053

[36] Hope WW, Demeter BL, Newcomb WL, Schmelzer TM, Schiffern LM, Heniford BT (2007) u. a. postoperative pulmonary embolism: timing, diagnosis, treatment, and outcomes. Am J Surg 1 Dezember 194(6):814–819 10.1016/j.amjsurg.2007.08.014 18005777

[37] Bjørnar̊ BT, Gudmundsen TE, Dahl OE (2006) Frequency and timing of clinical venous thromboembolism after major joint surgery. J Bone Joint Surg Br Volume März 88–B(3):386–391 10.1302/0301-620X.88B3.17207 16498018

[38] Sing RF, Camp SM, Heniford BT, Rutherford EJ, Dix S, Reilly PM (2006) u. a. timing of Pulmonary Emboli after Trauma: implications for Retrievable Vena Cava Filters. J Trauma Acute Care Surg April 60(4):732–735 10.1097/01.ta.0000210285.22571.66 16612291

[39] Anderson MC, Shields TW, SIGNIFICANCE OF FATAL PULMONARY, EMBOLISM IN IMMEDIATE POSTOPERATIVE PERIOD (1958) J Am Med Association 24 Mai 167(4):422–426 10.1001/jama.1958.02990210008002 13538716

[40] Konstantinides SV, Meyer G, Becattini C, Bueno H, Geersing GJ, Harjola VP (2020) u. a. 2019 ESC guidelines for the diagnosis and management of acute pulmonary embolism developed in collaboration with the European Respiratory Society (ERS): the Task Force for the diagnosis and management of acute pulmonary embolism of the European Society of Cardiology (ESC). Eur Heart J 21 Januar 41(4):543–603 10.1093/eurheartj/ehz405 31504429

[41] Martino MA, Borges E, Williamson E, Siegfried S, Cantor AB, Lancaster J (2006) u. a. pulmonary embolism after major abdominal surgery in Gynecologic Oncology. Obstet Gynecol März 107(3):666–671 10.1097/01.AOG.0000200046.28199.ae 16507939

[42] Memtsoudis SG, Besculides MC, Gaber L, Liu S, González Della Valle A (2009) Risk factors for pulmonary embolism after hip and knee arthroplasty: a population-based study. Int Orthop Dezember 33(6):1739–1745 10.1007/s00264-008-0659-z PMC2899166 18925395

[43] Paloncy R, Greimel F, Grifka J (2022) Ambulante Versorgung durch sektorübergreifende Prähabilitations- Und Rehabilitationskonzepte in Der tagesstationären Hüft- Und Kniegelenkendoprothetik. Orthopade 51(5):385–394 35441878 10.1007/s00132-022-04241-w PMC9019805

[44] Wente MN, Veit JA, Bassi C, Dervenis C, Fingerhut A, Gouma DJ (2007) u. a. postpancreatectomy hemorrhage (PPH): an International Study Group of pancreatic surgery (ISGPS) definition. Surg Juli 142(1):20–25 10.1016/j.surg.2007.02.001 17629996

[45] Correa-Gallego C, Brennan MF, D’Angelica MI, DeMatteo RP, Fong Y, Kingham TP (2012) u. a. contemporary experience with postpancreatectomy hemorrhage: results of 1,122 patients resected between 2006 and 2011. J Am Coll Surg November 215(5):616–621 10.1016/j.jamcollsurg.2012.07.010 22921325

[46] Mischinger HJ, Werkgartner G, Kornprat P, Marsoner K, Wagner D, Cerwenka H (2018) u. a. Komplikationen in Der Pankreaschirurgie. Wien Klin Mag 1 Juni 21(3):98–107

[47] Wellner UF, Kulemann B, Lapshyn H, Hoeppner J, Sick O, Makowiec F (2014) u. a. postpancreatectomy hemorrhage–incidence, treatment, and risk factors in over 1,000 pancreatic resections. J Gastrointest Surg März 18(3):464–475 10.1007/s11605-013-2437-5 24448997

[48] der Marckmann G (2011) Schmitten J. Wie können Ärzte ethisch vertretbar Kostenerwägungen in ihren Behandlungsentscheidungen berücksichtigen? Ein Stufenmodell. Ethik Med. 1. Dezember 23(4):303–14

[49] Hiller M, Spohn K, Schütte JK, Bracht H, Hering L, Bakker J Objektive Verlegungskriterien und proaktives Verlegungs­management zur Steuerung von intensivmedizinischen Kapazitäten. Hiller M, Spohn K, Schütte JK, Bracht H, Hering R, Bakker J: Objektive Verlegungskriterien und proaktives Verlegungsmanagement zur Steuerung von intensivmedizinischen Kapazitäten. 1. Dezember 2020;(12–2020):569–78

[50] Janssens U (2015) Ökonomie in Der Intensivmedizin – Ein Widerspruch? Med Klin Intensivmed Notfmed. 1 Mai 110(4):264–271 10.1007/s00063-015-0028-9 25917183

[51] Arumugam S, El-Menyar A, Al-Hassani A, Strandvik G, Asim M, Mekkodithal A (2017) u. a. delirium in the Intensive Care Unit. J Emerg Trauma Shock 10(1):37–46 28243012 10.4103/0974-2700.199520 PMC5316795

[52] Stollings JL, Kotfis K, Chanques G, Pun BT, Pandharipande PP, Ely EW (2021) Delirium in critical illness: clinical manifestations, outcomes, and management. Intensive Care Med 1 Oktober 47(10):1089–1103 10.1007/s00134-021-06503-1 PMC8366492 34401939

[53] Kotfis K, van Diem-Zaal I, Williams Roberson S, van den Sietnicki M, Shehabi Y (2022) u. a. The future of intensive care: delirium should no longer be an issue. Crit Care 5 Juli 26(1):200 10.1186/s13054-022-04077-y PMC9254432 35790979

