# Correction to: Prevalence of relevant early complications during the first 24 h on a normal ward in patients following PACU care after medium and major surgery: a monocentric retrospective observational study

**DOI:** 10.1007/s00423-024-03500-y

**Published:** 2024-10-22

**Authors:** Anouk Wurth, Thilo Hackert, Dittmar Böckler, Manuel Feisst, Sabine Haag, Markus A. Weigand, Thorsten Brenner, Thomas Schmoch

**Affiliations:** 1 Department of Anesthesiology and Intensive Care Medicine, University Hospital Essen, University Duisburg–Essen, Hufelandstraße 55, 45147 Essen, Germany; 2 Department of Anesthesiology, Heidelberg University Hospital, Heidelberg, Germany; 3 Department of General, Visceral and Transplantation Surgery, University of Heidelberg, Heidelberg, Germany; 4 Department of General, Visceral and Thoracic Surgery, University Hospital Hamburg–Eppendorf, Hamburg, Germany; 5 Department of Vascular Surgery and Endovascular Surgery, Heidelberg University Hospital, Heidelberg, Germany; 6 Institute of Medical Biometry, University of Heidelberg, Heidelberg, Germany; 7 Department of Anesthesiology and Intensive Care Medicine, Hôpitaux Robert Schuman – Hôpital Kirchberg, Luxembourg City, Luxembourg

**Correction to: Langenbeck’s Archives of Surgery (2024) 409:293**

10.1007/s00423-024-03480-z

This article unfortunately contained a mistake. An error was inadvertently introduced into the methodology section of the abstract during the production phase. Enumerations were replaced by citation numbers, which were then replaced by full citations, rendering the abstract unreadable.

**Methods** In this monocentric retrospective observational study, all adult patients who (Deutsche Gesellschaft für Anästhesiologie und Intensivmedizin (DGAI) in Anästh Intensivmed (50):S486–S489, 2009) underwent medium or major surgery between 1 January 1 2014 and 31 December 2018 at the Heidelberg University Surgical Center, and (Vimlati et al. in Eur J Anaesthesiol September 26(9):715–721, 2009) were monitored for 1–12 h in the PACU, and then (De Pietri et al. in World J Gastroenterol 20(9):2304–23207, 2014) transferred to a normal ward (NW) immediately thereafter were included. At the end of the PACU stay, each patient was cleared by both a surgeon and an anesthesiologist to be transferred to a NW. The first objective of this study was to determine the prevalence of relevant early complications (RECs) within the first 24 h on a normal ward. The secondary objective was to determine the prevalence of RECs in the subgroup of included patients who underwent partial pancreaticoduodenectomy.

should have read:

**Methods** In this monocentric retrospective observational study, all adult patients who (I) underwent medium or major surgery between 1 January 1 2014 and 31 December 2018 at the Heidelberg University Surgical Center, and (II) were monitored for 1–12 h in the PACU, and then (III) transferred to a normal ward (NW) immediately thereafter were included. At the end of the PACU stay, each patient was cleared by both a surgeon and an anesthesiologist to be transferred to a NW. The first objective of this study was to determine the prevalence of relevant early complications (RECs) within the first 24 h on a normal ward. The secondary objective was to determine the prevalence of RECs in the subgroup of included patients who underwent partial pancreaticoduodenectomy.

The original article has been corrected.

