# Supplementary material for: Prevalence of relevant early complications during the first 24 h on a normal ward in patients following PACU care after medium and major surgery: a monocentric retrospective observational study
Source: Langenbecks Arch Surg. 2024 Sep 30;409(1):293. doi: 10.1007/s00423-024-03480-z (PMC11442648; doi:10.1007/s00423-024-03480-z)
Supplement: Supplementary file 1 — Supplementary Material 1 [file 423_2024_3480_MOESM1_ESM.docx]

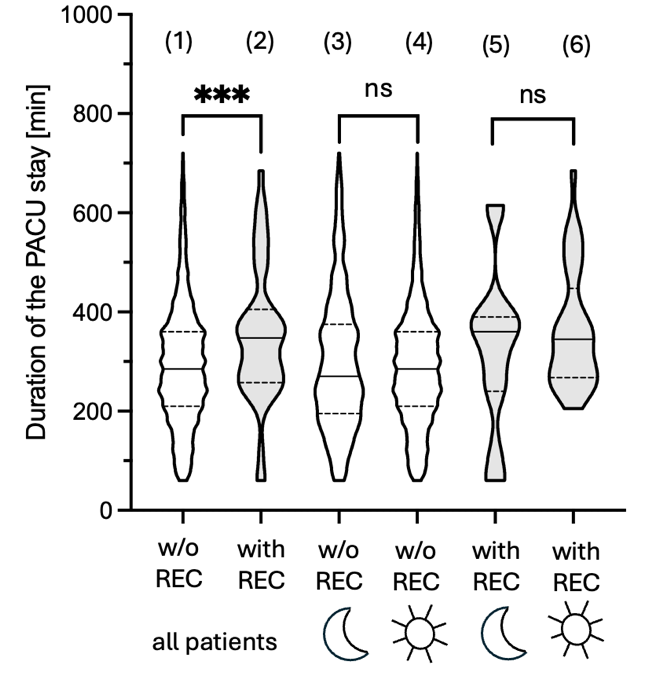


|  | Without REC | With  REC | Night arrival without REC | Day arrival without REC | Night arrival with REC | Day arrival with REC |
| --- | --- | --- | --- | --- | --- | --- |
| Number of values | 10225 | 48 | 1243 of 10225  (12.2%) | 8982 of 10225  (87.8%) | 15 of 48  (31.2%) | 33 of 48  (68.8%) |
| Duration of PACU stay, min |  |  |  |  |  |  |
| Minimum | 60.0 | 60.0 | 60.0 | 60.0 | 60.0 | 205.0 |
| 25th percentile | 210.0 | 257.5 | 195.0 | 210.0 | 240.0 | 267.5 |
| Median | 285.0 | 347.5 | 270.0 | 285.0 | 360.0 | 345.0 |
| 75th percentile | 360.0 | 405.0 | 375.0 | 360.0 | 390.0 | 447.5 |
| Maximum | 720.0 | 685.0 | 720.0 | 720.0 | 615.0 | 685.0 |
| Range | 660.0 | 625.0 | 660.0 | 660.0 | 555.0 | 480.0 |
|  |  |  |  |  |  |  |
| Mean | 292.7 | 351.4 | 297.2 | 289.9 | 323.0 | 364.2 |
| Std. deviation | 122.6 | 135.2 | 138.1 | 120.7 | 164.8 | 120.1 |
| Std. error of mean | 1.2 | 19.5 | 3.9 | 1.3 | 42.6 | 20.9 |

**Figure SI 1.** Comparison of durations of PACU stay**.**

Violin plots of the length of PACU stay values. Solid lines symbolize medians, dashed lines symbolize the 25th and 75th percentiles. The exact values can be found in the corresponding table below. From left to right, length of PACU stay statistics are shown as violin plots for the following patient groups:

(1) All patients without RECs.

(2) All patients with RECs.

(3) All patients without (w/o) RECs who were admitted to the recovery room at night (7 pm–6:55 am, moon symbol).

(4) All patients without (w/o) RECs who were admitted to the PACU during the day (7 am–6:55 pm, sun symbol).

(5) All patients with RECs who were admitted to the PACU at night (7 pm–6:55 am, moon symbol).

(4) All patients with RECs who were admitted to the PACU during the day (7 am–6:55 pm, sun symbol).

***Comparison of (1) and (2), p = 0.001; comparison of (3) and (4), not significant (ns); comparison of (5) and (6), not significant. PACU—post operative care unit; REC—relevant early complication; Std.—standard; w/o—without.

******

**Figure SI 2.** Violin plots of times of transfers from the PACU to the normal ward.

Violin plots of the times of transfers from the PACU to the normal ward. Solid lines symbolize medians, dashed lines symbolize the 25th and 75th percentiles. PACU—post operative care unit; REC—relevant early complication; w/o—without.

| **Year** | **2014** | **2015** | **2016** | **2017** | **2018** | **Total** | **Average** |
| --- | --- | --- | --- | --- | --- | --- | --- |
| Total number of patients, n | 7,919 | 8,306 | 8,157 | 8,477 | 8,442 | 41,301 | 8,260 |
| Share of medium and major surgery, % | 69.1 | 74.2 | 82.2 | 84.1 | 91.1 | n.a. | 80.2 |
| Included patients, n | 1,988 | 1,977 | 2,035 | 2,157 | 2,116 | 10,273 | 2,055 |
| Included patients, % | 25.1 | 23.8 | 24.9 | 25.4 | 25.1 | n.a. | 24.9 |
| REC, n | 12 | 10 | 10 | 3 | 13 | 48 | 9,6 |
| Admissions 7.00- a.m. - 6.55 p.m., n | 5,592 | 5,786 | 5,638 | 5,679 | 5,675 | 28,370 | 5,674 |
| Admissions 7.00- p.m. - 6.55 a.m., n | 1,073 | 1,083 | 1,009 | 1,178 | 1,199 | 5,542 | 1,108 |
| Emergency admissions 7.00- a.m. - 6.55 p.m, n | 675 | 637 | 617 | 630 | 614 | 3,173 | 635 |
| Emergency admissions 7.00- p.m. - 6.55 a.m., n | 412 | 426 | 389 | 454 | 409 | 2,090 | 418 |
| Total emergency admissions, n | 1,087 | 1,063 | 1,006 | 1,084 | 1,023 | 5,263 | 1,053 |
| Percentage of  ventilation, % | 5.0 | 6.8 | 7.6 | 14.6 | 5.8 | n.a. | 8.0 |

**Table SI 1:** Overview of the of PACU admission characteristics in the years 2014–2018

PACU—Post Anesthesia Care Unit; REC—relevant early complication

**Table SI 2:** Overview of patient distributions across operating departments.

|  | **Total**  n (%) | | **Without REC**  n (%) | | **With REC**  n (%) | | **Prevalence of REC**  (%) |
| --- | --- | --- | --- | --- | --- | --- | --- |
|  | **10,273** |  | **10,225** | (99.5) | **48** | (0.5) |  |
| **Operating specialty** |  |  |  |  |  |  |  |
| General, Visceral, and Transplant Surgery | 6,869 | (66.9) | 6,833 | (66.8) | 36 | (75.0) | 0.59 (CI 0.38–0.72) |
| Vascular Surgery | 1,184 | (11.5) | 1,177 | (11.5) | 7 | (14.6) | 0.60 (CI 0.29–1.22) |
| Urology | 2,220 | (21.6) | 2,215 | (21.7) | 5 | (10.4) | 0.23 (CI 0.10–0.53) |

CI—confidence interval; REC—relevant early complication.

**Table SI 3.** Overview of procedures coded in general and visceral surgery.

| **Organ system operated on, n (%)** | **6,869** | **(100.0)** |
| --- | --- | --- |
| Intestine* | 1,981 | (28.8) |
| Pancreas | 1,547 | (22.5) |
| Liver Interventions | 885 | (12.9) |
| Stomach | 403 | (5.9) |
| Gastric surgeries | 47 | (0.7) |
| Exploratory laparotomies | 648 | (9.4) |
| Diagnostic laparoscopy (peritoneoscopy) | 147 | (2.1) |
| Other abdomen | 414 | (6.0) |
| Thorax | 39 | (0.6) |
| Esophagus | 28 | (0.4) |
| Major urogenital and gynecological surgeries | 357 | (5.2) |
| Operations on endocrine glands including adrenalectomy | 91 | (1.3) |
| Major traumatological operations | 28 | (0.4) |
| Other | 254 | (3.7) |

* Note: According to the stated inclusion and exclusion criteria patients undergoing low risk procedures such as appendectomy were not included in the work.

**Table SI 4:** Overview of procedures coded as vascular surgery.

| **Operation type n (%)** | **1,184** | **(100.0)** |
| --- | --- | --- |
| Incision, excision, and closure of blood vessels | 869 | (73.4) |
| Creation of shunts and bypasses on large blood vessels | 170 | (14.4) |
| Proximal amputations | 71 | (6.0) |
| Abdominal surgery | 20 | (1.7) |
| Patch sculptures | 4 | (0.3) |
| Transposition of blood vessels | 4 | (0.3) |
| Revision of a blood vessel operation | 24 | (2.0) |
| Other | 22 | (1.9) |

**Table SI 5.** Overview of the most frequent procedures coded as urology.

| **Organ system operated on n (%)** | **2,220** | **(100.0)** |
| --- | --- | --- |
| Prostate and vesiculae seminales | 1,307 | 58.9 |
| Kidney | 485 | 21.8 |
| Ureter | 127 | 5.7 |
| Cystectomy | 20 | 0.9 |
| Other surgeries on urinary organs | 11 | 0.5 |
| Exenteration (evisceration) of the female lesser pelvis | 7 | 0.3 |
| Gastrointestinal tract | 50 | 2.3 |
| Other | 213 | 9.6 |

**Table SI 6:** Age stratification of the patients

|  | **Total**  n (%) | | **Without REC**  n (%) | | **With REC**  n (%) | |
| --- | --- | --- | --- | --- | --- | --- |
|  | **10,273** |  | **10,225** | 99.5% | **48** | 0.5% |
|  |  |  |  |  |  |  |
| **Age** (years) |  |  |  |  |  |  |
| 18–29 | 394 | (3.8) | 392 | (3.8) | 2 | (4.2) |
| 30–39 | 542 | (5.3) | 540 | (5.3) | 2 | (4.2) |
| 40–49 | 969 | (9.4) | 969 | (9.4) | 0 | (0.0) |
| 50–59 | 2275 | (22.1) | 2261 | (22.1) | 14 | (29.2) |
| 60–69 | 3123 | (30.4) | 3108 | (30.4) | 15 | (31.3) |
| 70–79 | 2453 | (23.9) | 2440 | (23.9) | 13 | (27.1) |
| 80–89 | 491 | (4.8) | 489 | (4.8) | 2 | (4.2) |
| 90–100 | 26 | (0.3) | 26 | (0.3) | 0 | (0.0) |
|  |  |  |  |  |  |  |

REC—relevant early complication.

**Table SI 7:** Distribution of Revised Cardiac Risk Index scores aross operating departments

|  | **General Surgery** | | | | **Vascular Surgery** | | | | **Urology** | | | |
| --- | --- | --- | --- | --- | --- | --- | --- | --- | --- | --- | --- | --- |
| **RCRI** | Without REC  **n (%)** | | With REC  **n (%)** | | Without REC  **n (%)** | | With REC  **n (%)** | | Without REC  **n (%)** | | With REC  **n (%)** | |
|  | 6,833 | (100) | 36 | (100) | 1,177 | (100) | 7 | (100) | 2,215 | (100) | 5 | (100) |
| **RCRI = 0** | 5,578 | (81.6) | 28 | (77.8) | 608 | (51.7) | 5 | (71.4 | 1,868 | (84.3) | 4 | (80.0) |
| **RCRI = 1** | 1,019 | (14.9) | 5 | (13.9) | 390 | (33.1) | 0 | (0.0) | 300 | (13.5) | 1 | (20.0) |
| **RCRI = 2** | 206 | (3.0) | 1 | (2.8) | 127 | (10.8) | 2 | (28.6) | 40 | (1.8) |  | (0.0) |
| **RCRI = 3** | 27 | (0.4) | 2 | (5.6) | 45 | (3.8) | 0 | (0.0) | 7 | (0.3) |  | (0.0) |
| **RCRI = 4** | 3 | (0.0) | 0 | (0.0) | 7 | (0.6) | 0 | (0.0) | 0 | (0.0) |  | (0.0) |

RCRI—Revised Cardiac Risk Index; REC—relevant early complication.

**Table SI 8:** Operations with relevant early complications in general surgery

| **Operation** | **OPS** | **Complication (n)** |
| --- | --- | --- |
| **Diagnostic laparoscopy** | 1-694 K | - Post-operative bleeding (1) |
| **Small bowel partial resection** | 5-454.X | - Tachyarrhythmia absoluta, with atrial fibrillation already known before surgery (1) - Respiratory decompensation (1) |
| **Partial colon resections** |  |  |
| Sigmoid resection | 5-455.72 | - Ischemia of the descendostoma (1) - Non-ST-elevation myocardial infarction (1) |
| Colectomy (open) with ileoanal anastomosis | 5-456.03 | - Post-operative bleeding (1) |
| Adhesiolysis of the bowel (open) | 5-469.20 | - Respiratory decompensation (1) - Pulmonary artery embolism (1) |
| **Laparoscopic proctocolectomy** | 5-456.15 | - Post-operative bleeding (1)) |
| **Open rectal resections** |  |  |
| Anterior rectum resection with sphincter preservation | 5-484.31 | - Post-operative bleeding (1) |
| Deep anterior rectal resection with descendorectostomy | 5-484.51 | - Post-operative bleeding and delirium (1) |
| Rectal resection with sphincter preservation | 5-484.52 K | - Respiratory decompensation (1) |
| **Liver surgery** |  |  |
| Pericystectomy of the liver, open | 5-501.20 | - Bile leakage (1) |
| Bisegmentectomy (lobectomy left) | 5-502.1 | - Pulmonary artery embolism (exitus letalis) (1) |
| Resection liver segments VI/VII + atype V | 5-502.2 | - Transaminase increase without evidence of ischemia (1) |
| Biliodigestive anastomosis | 5-512.1 | - Syncope during initial mobilization (1) |
| Excision and resection of diseased tissue | 5-515.2 | - Transaminase increase without evidence of ischemia (1) |
| Duodenotomy, papillary excision | 5-518.1X | - Tachyarrythmia absoluta in pre-existing atrial fibrillation (1). |
| Cholecystectomy in the course of exploratory laparotomy | 5-511.42 | - Acute renal failure (1) |
| **Pancreas operations** |  |  |
| Exploration, multivisceral resection if necessary; partial resection of the pancreas: pancreatic tail resection, open surgical | 5-524.00 | - Psychogenic decompensation (1) - Respiratory decompensation at initial mobilization to bedside. Patient with known OSAS (with home CPAP device) (1) - Post-operative bleeding (1) |
| Pancreatic left resection | 5-524.01 | - Pulmonary artery embolism |
| Whipple procedure (non-pylorus-preserving) | 5-524.1 | - Pulmonary artery embolism (1) - Syncope during initial mobilization (1) - Insufficiency of the bilio-digestive anastomosis (1) - Post-operative bleeding (1) |
| Whipple (pylorus-preserving) | 5-524.2 | - Pulmonary artery embolism (2) - Post-operative bleeding (1) - Insufficiency of the biliodigestive anastomosis (1) |
| Laparoscopy with drainage (DaVinci pancreatic left resection + splenectomy) | 5-549.5 | - Post-operative bleeding (1) |
| Exploratory laparotomy with biopsies | 5-541.0 | - Post-operative bleeding (1) - Respiratory decompensation (1) |
| Tumor resection (retropancreatic) | 5-590.51 | - Acute renal failure (1) |
| Resection of tissue, without certain organ affiliation | 5-590.81 | - Pain exacerbation (1) |

CPAP—continuous positive airway pressure; OPS—Operation and Procedure Code of the International Classification of Procedures in Medicine; OSAS—obstructive sleep apnea syndrome;

**Table SI 9:** Operations with relevant early complications in vascular surgery

| **Operation** | **OPS** | **Complication (n)** |
| --- | --- | --- |
| Abdominal aorta: bifurcation prosthesis | 5-38A.14 | - Post-operative bleeding (1) |
| Creation of a femoro-popliteal bypass | 5-393.54 | - Ischemia compartment formation (1) |
| Femoral-Crural bypass | 5-393.55 | - Ischemia in bypass occlusion (1) |
| Shunt thrombectomy upper arm | 5-394.5 | - Shunt closure (1) |
| Re-percutaneous transluminal angioplasty of the Left Superficial Femoral Artery | 8-836.0B | - Respiratory decompensation (1x) |
| Bypass thrombectomy | 5-394.1 | - Ischemia in bypass occlusion (1) |
| Endarterectomy thigh | 5-381.71 | - Re-ischemia (1) |

**Table SI 10:** Surgeries with relevant early complications in urology

| **Operation** | **OPS** | **Complication** |
| --- | --- | --- |
| Partial kidney resection | 5-553.00 | - Hypertensive emergency and delirium (1) |
| Da Vinci prostatectomy | 5-604.42 | - Post-operative bleeding (1) |
| Da Vinci prostatectomy | 5-604.52 | - Post-operative bleeding (2) - Syncope (1) |

OPS—Operation and Procedure Code of the International Classification of Procedures in Medicine

**List SI1 Extraction - Standard Operating Procedure “Early transfer PACU”**

**Checklist of transfer criteria:**

- Medium arterial pressure (MAP) >65 mmHg
- Heart rate <100/min
- Diuresis >1 ml / kg body weight / hour
- Hemoglobin >8 g/dl (no significant dynamics in the last 4h)
- Drainage output: serous (not bloody); <1000ml since surgery; and no significant dynamics in the last 4 h
- Quick > 40% (without substitution in the PACU)
- Base Excess > –4
- No significant fluid requirement in the last 4 hours
- Normoglycemia (no continuous insulin requirement; Glycemia >100 mg/dl)
- Saturation: >95% (with less than 4L O_2_ via nasal probe)
- Neurology without pathological findings
- Good pain compensation under standard analgesia
